# P-654. Health-related Quality of Life Associated with Pneumococcal Disease – A Global Targeted Literature Review and Meta-Analysis

**DOI:** 10.1093/ofid/ofae631.851

**Published:** 2025-01-29

**Authors:** Min Huang, Hela Romdhani, Jipan Xie, Yan Song, Sun Lee, Daisy Liu, Elamin Elbasha, Salini Mohanty, Donna Rowen, Matthew S Kelly

**Affiliations:** Merck & Co., Inc., Rahway, New Jersey; Analysis Group, Inc., Boston, Massachusetts; XL Source, Inc., Los Angeles, California; Analysis Group, Inc., Boston, Massachusetts, USA, Boston, MA; Analysis Group, Inc., Boston, Massachusetts; Analysis Group, Inc., Boston, Massachusetts; Merck & Co., Inc., Rahway, New Jersey; Merck & Co., Inc, Rahway, New Jersey; University of Sheffield, Sheffield, England, United Kingdom; Duke University School of Medicine, Durham, NC

## Abstract

**Background:**

Pneumococcal disease (PD) is a group of infections that can have a significant yet poorly understood impact on health-related quality of life (HRQoL). This meta-analysis aims to quantify the HRQoL impact of PD.

**Methods:**

Original research studies reporting health utility, disutility, quality-adjusted life year (QALY), or QALY decrement associated with PD were identified through a global targeted literature review in MEDLINE (March 2023). Health utilities and QALYs were converted to QALY decrements per episode using reported or imputed duration of illness. QALY decrements for different PD categories were estimated using random effects models, with heterogeneity assessed using τ^2^ and I^2^ statistics.

**Results:**

The literature review identified 27 original utility studies of PD from 19 countries published in 1993-2021. These studies used various utility estimation methods, including direct methods (e.g., standard gamble, time trade-off, visual analogue scale) and indirect methods (e.g., EQ-5D, Health Utilities Index). Estimates (95% confidence intervals) of QALY decrement per episode derived from the meta-analyses in adults (≥18 years) were 0.033 (0.027, 0.040) for meningitis, 0.019 (0.011, 0.027) for bacteremia/sepsis, 0.018 (0.011, 0.024) for inpatient pneumonia, and 0.007 (0.006, 0.009) for outpatient pneumonia. The corresponding values in children (0-17 years) were 0.029 (0.019, 0.039), 0.016 (0.008, 0.024), 0.026 (0.010, 0.042), and 0.013 (0.007, 0.020). The estimates for acute otitis media (AOM) and recurrent AOM in children were 0.004 (0.002, 0.006) and 0.007 (0.003, 0.010), respectively. Significant heterogeneity was observed across studies for nearly all PD categories, largely due to variations in methods.

**Conclusion:**

PD has a substantial negative impact on HRQoL in children and adults. The extent of impairment varies widely across PD categories, with the greatest impairment observed in meningitis, bacteremia/sepsis, and inpatient pneumonia. The review reveals significant heterogeneity in methods and results across studies. Studies incorporating more recent data and using standard methods are needed to accurately estimate the impact of PD on HRQoL and inform cost-effectiveness analyses of vaccines that prevent PD.

**Disclosures:**

**Min Huang, PhD**, Merck & Co., Inc.: Employee|Merck & Co., Inc.: Stocks/Bonds (Public Company) **Hela Romdhani, PhD**, Analysis Group, Inc.: Employee|Merck & Co., Inc.: I am an employee of Analysis Group, Inc., a consulting company hat has provided paid consulting services to Merck & Co., Inc. **Jipan Xie, MD, PhD**, XL Source, Inc.: Jipan Xie is an employee of XL Source, Inc., which received research funding from Analysis Group, Inc. for this study. **Yan Song, PhD**, Analysis Group, Inc.: Employee|Merck & Co., Inc.: I am an employee of Analysis Group, Inc., a consulting company hat has provided paid consulting services to Merck & Co., Inc. **Sun Lee, PharmD, MPH**, Analysis Group, Inc.: Employee|Merck & Co., Inc.: I am an employee of Analysis Group, Inc., a consulting company hat has provided paid consulting services to Merck & Co., Inc. **Daisy Liu, MS**, Analysis Group, Inc.: Employee|Merck & Co., Inc.: I am an employee of Analysis Group, Inc., a consulting company hat has provided paid consulting services to Merck & Co., Inc. **Elamin Elbasha, Ph.D.**, Merck & Co., Inc.: Employee|Merck & Co., Inc.: Stocks/Bonds (Public Company) **Salini Mohanty, DrPH, MPH**, Merck & Co., Inc.: Employee|Merck & Co., Inc.: Stocks/Bonds (Public Company) **Donna Rowen, PhD**, Costello: I have received financial payment in return for advice or training on utility estimation.|Merck & Co., Inc.: I have received financial payment in return for advice or training on utility estimation.|Novartis: I have received financial payment in return for advice or training on utility estimation.|Novo Nordisk: I have received financial payment in return for advice or training on utility estimation.|Sanofi: I have received financial payment in return for advice or training on utility estimation. **Matthew S. Kelly, MD, MPH**, Invivyd: Advisor/Consultant|Merck & Co., Inc.: Advisor/Consultant|Merck & Co., Inc.: I am an employee of Duke University Medical Center, which received grant funding from Merck & Co., Inc.

